# Time to Look Beyond Age? Frailty as the Key Determinant of EVT Prognosis in Acute Ischemic Stroke Patients ≥75 Years

**DOI:** 10.1093/geroni/igaf122.4336

**Published:** 2025-12-31

**Authors:** Wenhua Yu, Lina Ma

**Affiliations:** Xuanwu Hospital, Capital Medical University, Beijing, Beijing, China (People’s Republic); Xuanwu Hospital, Capital Medical University, Beijing, Beijing, China (People’s Republic)

## Abstract

**Background:**

Stroke is a major public health concern worldwide and the primary cause of adult mortality in China. Endovascular treatment (EVT) has been increasingly utilized in older acute ischemic stroke (AIS) patients; however, there is limited data regarding the association between frailty and prognosis after EVT in individuals aged ≥75 years.

**Objective:**

To evaluate frailty assessment as a prognostic indicator following EVT in patients with AIS aged ≥75 years.

**Methods:**

We included 193 patients with AIS aged ≥75 years who underwent EVT at Xuanwu Hospital of Capital Medical University from May 2013 to July 2023. Frailty was assessed using the Clinical Frailty Scale. The primary outcome was defined as a favorable modified Rankin Scale score of 0–1 at 3 months after EVT. We used multivariable logistic regression to evaluate the impact of frailty on clinical outcomes.

**Results:**

The mean age of participants was 80.34±4.39 years. Frailty, older age, female sex, consumed less alcohol, stroke history, coronary heart disease, more chronic diseases, lower Barthel Index, higher admitted National Institutes of Health Stroke Scale scores, higher fasting blood glucose levels, higher neutrophil percentage, higher D-Dimer levels and lower hemoglobin levels were associated with unfavorable outcomes. Logistic regression analysis revealed that frailty and stroke history were associated with poor prognosis after EVT in older adults with AIS.

**Conclusions:**

Frailty is an indicator of poor prognosis following EVT in patients with AIS aged ≥75 years. Frailty assessment provides valuable insights for clinical decision-making, helping guide more informed EVT decisions in this age group.

